# Noninvasive low-level tragus stimulation attenuates inflammation and oxidative stress in acute heart failure

**DOI:** 10.21203/rs.3.rs-3323086/v1

**Published:** 2023-09-11

**Authors:** Tarun Dasari, Praloy Chakraborty, Peter Mukli, Khawaja Akhtar, Andriy Yabluchanskiy, Madeleine W. Cunningham, Anna Csiszar, Sunny S. Po

**Affiliations:** University of Oklahoma: The University of Oklahoma; University of Oklahoma: The University of Oklahoma; University of Oklahoma: The University of Oklahoma; University of Oklahoma: The University of Oklahoma; University of Oklahoma: The University of Oklahoma; University of Oklahoma: The University of Oklahoma; University of Oklahoma: The University of Oklahoma; University of Oklahoma: The University of Oklahoma

**Keywords:** Low-level stimulation of the tragus, acute heart failure, inflammation, oxidative stress, sympathovagal balance

## Abstract

**Purpose:**

Acute decompensated heart failure is associated with inflammation, oxidative stress, and excess sympathetic drive. It is unknown if neuromodulation would improve inflammation and oxidative stress in acute heart failure. We, therefore, performed this proof-of-concept study to evaluate the effects of neuromodulation using noninvasive low-level Tragus stimulation on inflammation and oxidative stress in ADHF.

**Methods:**

19 patients with ejection fraction < 40% were randomized to neuromodulation- 4 hours twice daily (6 AM-10 AM and 6 PM-10 PM) (n = 8) or sham stimulation (n = 11) during hospital admission. All patients received standard-of-care treatment. Blood samples were collected at admission and discharge. Serum cytokines were assayed using standard immunosorbent techniques. Reactive oxygen species inducibility from cultured coronary endothelial cells exposed to patient sera was determined using dihydrodichlorofluorescein probe test (expressed as fluorescein units).

**Results:**

Compared to sham stimulation, neuromodulation was associated with a significant reduction of circulating serum Interleukin-6 levels (−78% vs −9%; p = 0.012). Similarly, neuromodulation led to reduction of endothelial cell oxidative stress, in the neuromodulation group (1363 units to 978 units, p = 0.003) compared to sham stimulation (1146 units to 1083 units, p = 0.094). No significant difference in heart rate, blood pressure or renal function were noted between the two groups.

**Conclusion:**

In this proof-of-concept pilot study, in acute systolic heart failure, neuromodulation was feasible and safe and was associated with a reduction in systemic inflammation and attenuation of cellular oxidative stress.

**Clinical trial:**

NCT02898181

## Introduction

Acute decompensated heart failure (ADHF) is the most common cause of hospitalization in patients with heart failure.^1^ Despite major advances in the management of chronic heart failure, limited treatment options are available for ADHF patients. Pharmacological management with diuretics, correction of precipitating factors, and supportive measures including vasodilators or inotropes remain the mainstay for ADHF.^2^ Even with the most modernized treatment, ADHF carries poor outcome with recurrent hospitalizations and increased mortality.^2, 3^ Considering the burden of ADHF and the limitations of currently available therapies, there is an unmet need for novel treatment strategies for ADHF.

A dysregulated autonomic nervous system, characterized by higher sympathetic and blunted parasympathetic response is the hallmark of heart failure.^4^ Although the enhanced sympathetic activity helps to maintain the end organ perfusion, in the long term it is associated with progressive and irreversible deterioration of cardiac function. Pro-inflammatory cytokines and oxidative stress contribute to disease progression by inducing contractile dysfunction, cell death, apoptosis, and fibrosis.^5, 6^ β-adrenergic receptor mediated stimulation of intracellular cyclic adenosine monophosphate and subsequent downstream kinase activation is one principal cause of maladaptive contractile, metabolic, and structural remodeling of the ventricle in heart failure.^7^ Hemodynamic stress, neurohormonal activation, and hypoperfusion also contribute to deranged cardiac metabolism, abnormal cardiac redox state, and cardiac and systemic inflammation.

The tragus of the external ear is innervated by the auricular branch of the vagus nerve. Low-level tragus stimulation (LLTS) is an emerging noninvasive neuromodulation strategy that is found to be associated with beneficial remodeling of the cardiac autonomic nervous system and restoration of sympathovagal balance.^8–10^ LLTS is found to ameliorate inflammation, remodeling in heart failure with preserved ejection fraction.^9, 11, 12^ However, the role of LLTS in ADHF has not been evaluated. The current study evaluated the effects of LLTS on biomarkers of inflammation and oxidative stress in patients with ADHF. Here, we harnessed the beneficial effects of the neuro-immune axis to potentially reduce inflammation and oxidative stress in ADHF.

## Materials and methods

### Study protocol

This single-center, prospective, double-blind, randomized study was conducted at the University of Oklahoma Medical Center. Patients who were hospitalized with ADHF with left ventricular ejection fraction (LVEF) < 40% were recruited. Patients with any of the following criteria were excluded from the study: acute coronary syndrome, complex congenital heart disease, recurrent vasovagal syncope, history of unilateral or bilateral vagotomy, sick sinus syndrome, cardiac conduction abnormalities including 2nd, or 3rd -degree atrioventricular block, bifascicular block or prolonged 1st degree AV block (PR > 300ms), pregnancy, prisoners, or end-stage renal disease on dialysis. The study was approved by the local Institutional Review Board. After informed consent, included patients were randomly assigned (using a computer generated model allocation on REDCAP) to either active LLTS or sham stimulation which was continued for the duration of their index hospital admission. The baseline clinical data including physical examination, routine laboratory investigation, electrocardiography, and echocardiography data were recorded. Patients were then instructed about the process of using the device and were asked to repeat the process themselves under direct supervision, to ensure the correct use of the device. This demonstration process was performed by a clinical coordinator who was not blinded to random allocation. All other investigators were blinded to the allocation arm. Blood samples were collected on the day of admission before the initiation of the treatment protocol and on the day of discharge. All procedures and protocols were approved by the Institutional Review Board at the University of Oklahoma. Informed consent was obtained from each participant.

### Stimulation Protocol

Active LLTS was performed using electrodes attached to the tragus of the ear, which is innervated by the auricular branch of the vagus nerve.^13^ The PARASYM^®^ (Parasym Ltd, London, United Kingdom) TENS Unit, was used for LLTS. The device was connected to a clip electrode that was attached to the external ear. In the active group, the ear clip electrode was attached to the tragus. In the sham stimulation group, the clip electrode was attached to the ear lobule where no vagal innervation is present. The unit was set at a pulse width of 200 μs, and a pulse frequency of 20 Hz. These parameters were chosen based on our previous published work in diastolic dysfunction and paroxysmal atrial fibrillation. The amplitude was titrated to the level of sensory threshold, typically in the range of 10–50 mA. The stimulation strength was gradually increased until the patient experienced mild discomfort and was then decreased by 1mA below the threshold of stimulation. LLTS was applied for 8 hours daily (4 hours twice daily, between 6 AM-10 AM and 6 PM −10 PM), and the device was used throughout the index hospital admission.

### Serum inflammatory markers

Blood samples were centrifuged within 30 minutes of collection (4000g for 10 minutes), and serum/plasma was immediately stored in aliquots at −80°C. To determine systemic inflammation, serum levels of IL-6 were measured. As a proof of concept we also analyzed certain other markers in inflammation: CRP, TNFα, soluble intercellular adhesion molecule 1 (sICAM-1), and vascular cell adhesion molecule 1 (sVCAM-1) using commercially available enzyme linked immunosorbent assay kits (Thermo Fisher Scientific^™^, USA)

### Oxidative stress

Primary human coronary endothelial cells (HCAECs) based bioassay was used to evaluate the oxidative stress-inducing capacity of patient sera. Detector HCAECs were cultured in an Endothelial Basal Medium supplemented with 10% fetal calf serum until the time of serum treatment, as described.^14^ For treatment, fetal calf serum was replaced with patient sera (10%; for 48 h), following published protocols.^14^ Sera collected before and after treatment was distributed in a 96-well plate in quadruplicates, samples, and from each patient were measured in the same plate. Production of reactive oxygen species (ROS) by cultured HCAECs was measured by flow cytometry after treatment with sera of ADHF patients. Intracellular production of ROS induced by serum factors was measured in detector HCAECs using 2,7 dihydrodichlorofluorescein (DCF) at 3 micromol concentration.^15^

### Statistical Analysis:

The primary outcome of the study was the reduction in inflammatory activity and ROS (cytosolic peroxide content) during hospital admission. We did not perform power calculations in this first phase of feasibility trial. Categorical data were presented as percentages. For continuous variables, the distribution of data was assessed using the Shapiro-Wilk test and variance homogeneity with F-test. Normally distributed continuous data were presented as mean ± SD whereas median(IQR) values were used to present skewed data. The serum inflammatory and oxidative stress markers were compared between two groups (baseline LLTS vs baseline sham control or baseline vs predischarge) using the ANOVA test and within group changes were analyzed using Wilcoxon sum rank test at 5% significance.

## Results

### Study Population

20 patients were enrolled in the study, but 19 patients completed the study, and data from these 19 patients were used for analysis. Of these 19 patients, 8 patients (42%) were randomized to the intervention group, and 11 (52%) patients were randomized to the sham stimulation (control) group. The demographics and baseline characteristics of both groups are presented in Supplemental Table 1. No adverse events were reported by patients and device was well tolerated by all subjects. One patient left the hospital against medical advice and hence follow up data was not available.

### LLTS attenuates systemic inflammation:

**Figure 1:**
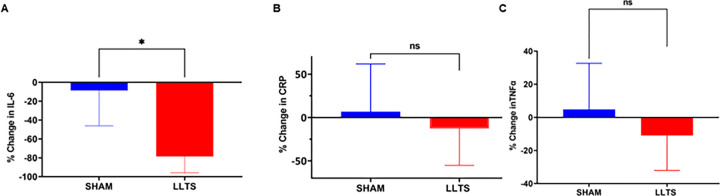
Effect of LLTS on serum inflammatory markers: Serum inflammatory cytokine levels were assessed using high sensitivity ELISA. Changes from baseline to discharge values were expressed as percentage of the baseline value (discharge-baseline/baseline × 100). Changes were compared between LLTS and control. *p<0.05, Wilcoxon-test

In Tables 1 and 2 inflammatory biomarker values recorded at baseline were compared to the values at discharge in both LLTS and control groups. In Table 3, differences are considered by evaluating the percent change between baseline and discharge. Changes from baseline to discharge value for each group and cytokines were expressed as percentage of baseline values. LLTS was associated with significant reduction in IL-6 level (−78.48% [53.42] vs −8.63% [32.80]; *p* = 0.012) (Fig. 1). Other inflammatory markers assayed: change in TNFα (−10.91% [41.46] vs 4.78% [31.98; *p*:0.182), CRP (−12.69% [60.28] vs 6.74% [84.06]; *p*:0.549), sICAM-1 (−2.82 [88.20] vs 2.29 [79.13]; p = 0.720) and sVCAM-1(−3.46 [45.17] vs 19.38 [19.29]; p = 0.400) levels were also favorably lowered by LLTS but when compared to sham control, but they did not reach statistical significance. (Table 3, Figs. 1).

### LLTS attenuates oxidative stress in human coronary endothelial cells

**Figure 2:**
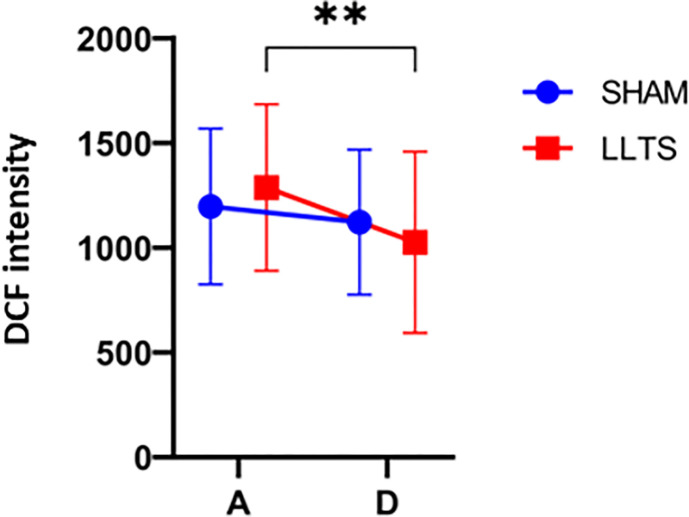
Effect of LLTS on oxidative stress: Flow cytometric analysis of oxidative stress markers in cultured human coronary endothelial cells (HCAEC) exposed to sera of patients with acute heart failure (AHF). Fluorescent intensity values (arbitrary units) in the lower-level tragus stimulation (LLTS) group are shown as red squares (n=8 for all panels), while data in the sham treatment group are shown as blue circles (n=10 for all panels). A: admission (first visit), D: discharge (second visit). The effect of LLTS/sham treatment on median dichlorodihydrofluorescein (DCF) fluorescence intensity [indicating intracellular reactive oxygen species (ROS) production] was significantly lower only after LLTS (**p<0.005, Wilcoxon-test).

Cultured HCAEC were exposed to the serum and subsequent measurement of the fluorescent intensity of reactive oxygen species using sensitive fluorescent probes 2,7-dichlorodihydrofluorescein (DCF). Results were expressed in fluorescein units. LLTS was associated with a significant reduction in pre-discharge DCF fluorescence (1363 [830.7] vs. 978.3 [735.3]; *p* = 0.004) (Table 1 and Fig. 2). However, sham stimulation was not associated with a significant change in DCF fluorescence (1146[528.7] vs 1083[586.20]; *p* = 0.10) (Table 2 and Fig. 2). Therefore, markers of oxidative stress were reduced significantly in the LLTS group.

## Discussion

Our first-in-human, proof-of-concept, pilot study demonstrated the feasibility and safety of LLTS in ADHF and showed improvement in systemic inflammation, as evident in IL6. Similarly, changes were noted in oxidative stress with LLTS compared to sham. LLTS did not adversely affect heart rate, blood pressure, and renal function during hospitalization. This is the first study to demonstrate favorable effects of LLTS on inflammation and oxidative stress in ADHF.

### Effects of LLTS on inflammation:

In our pilot, proof-of-concept study in acute heart failure, neuromodulation technique using LLTS was associated with a reduction in serum IL-6 but also favorably altered other cytokines (CRP, TNF-α). The reduction of systemic inflammation by LLTS in ADHF is a novel finding of our study. In addition, neuromodulation using LLTS led to favorable modulation of human coronary endothelial cell oxidative stress.

ADHF is considered a proinflammatory state and a significant increase in inflammatory cytokines (Il-6, CRP, TNF-α) is reported in hospitalized patients with ADHF.^16–18^ The acute increase in cytokines may modulate the course of acute heart failure by negatively influencing ventricular systolic and diastolic function, systemic and pulmonary fluid homeostasis, and promoting prothrombotic and proapoptotic milieu. Serum cytokine levels are also reported to correlate with the degree of ventricular remodeling and adverse prognosis.^16, 17^ IL-6 levels at admission are found to correlate with the severity of heart failure and mortality.^19–21^ IL-6, in acute myocarditis, also plays a crucial role in the transition from acute to chronic phase by promoting macrophage recruitment, and persistent elevation of IL-6 is known to predict the risk for recurrent hospitalization and non-recovery of ventricular function.^21 22^ The severity of myocardial damage and remodeling correlates with the degree of inflammation. In a canine model of ischemic heart failure, a greater reduction of inflammation with LLTS translated into more reverse remodeling and improvement of contractile function.^23^

Mechanisms underlying vagal-mediated inflammatory modulation have been described previously. In experimental models of systolic heart failure, restoration of sympathovagal balance by chronic vagal stimulation is associated with inhibition of cytokine release and improvement of ventricular function.^23, 24^ A vagally mediated ‘inflammatory reflex’ is known to control the immune response and inflammation during pathogenic invasion as well as during tissue injury, the parasympathetic vagal system being the integral component of both afferent and efferent system in this communication.^25^ LLTS is thought to relay afferent information to the dorsal vagal complex and dorsal motor nucleus through the auricular branch of the vagus nerve.^26^ Modulation of the brainstem stem vagal centers is thought to modulate the interaction between the sympathetic and parasympathetic systems with a net increase in efferent vagal outflow to the heart and periphery, specifically the splenic tissue. This anti-inflammatory reflex is partly mediated via α7 nicotinic acetylcholine receptor receptors (α7nAchR) on splenic macrophages thereby modulating the peripheral generation of inflammatory cytokines.^11, 27^ In summary, the vagomimetic and antiadrenergic action of LLTS, via the brain-cardiac-splenic axis, may explain the attenuation of inflammation in our study. LLTS-mediated reduction in systemic inflammation may suggest and uncover a novel, non-pharmacological intervention that may aid in treating a pathophysiological axis in ADHF.

### Effect of LLTS on oxidative stress:

Coronary endothelium is an important source of ROS generation in HF and our study demonstrated an attenuation of endothelial oxidative stress with neuromodulation via LLTS. Reactive oxygen species are primarily comprised of superoxide (O2^−^), hydroxyl (OH^−^) and peroxide(H_2_O_2_). These (specifically superoxide and hydroxyl) are principally generated in mitochondria and cytosol from electron leak through electron transport chain and oxidation of reduced nicotinamide adenine dinucleotide phosphate by its corresponding oxidase enzyme.^28^ Superoxide, an extremely unstable compound with half-life in nano seconds, is subsequently metabolized to H_2_O_2_ by superoxide dismutase. Due to rapid reduction by superoxide dismutase and very short half-life, superoxide is far more unstable compared to H_2_O_2_ and thereby rendering it as a less effective direct oxidative biomarker to measure. ^28^ Cytosolic peroxide is primarily dependent on diffusion of mitochondrial H2O2 into cytosol.^29, 30^

Dihydrodichlorofluorescein diacetate is widely used to evaluate cellular oxidative stress. DCF passes through plasma membrane and this non fluorescent compound is ultimately oxidized to a fluorescent compound dichlorofluroscein (DCF), via a process that involves various ROS, but more specific to H_2_O_2_.^31^ This oxidation is also heavily dependent on Cytochrome c (residing on the inner membrane of the mitochondria) and redox active transition metals.^32^ In a previous study by Csiszar et.al., endothelial cells exposed to increased inflammatory stress (such as increased circulating cytokines-TNF- α) with subsequent increased oxidative stress and ROS generation, specifically peroxide, led to higher DCF values.^30, 33^ Increased circulating cytokines perhaps exposed the HCAEC’s to an inflammatory milieu leading to increased ROS generation and subsequent increased DCF fluorescence. To our knowledge, this is the first study in ADHF to determine the extent of induced human coronary endothelial ROS quantification, using DCF based assays, specific for cytosolic peroxide content.

An aberrant redox balance, characterized by enhanced pro-oxidant ROS generation and /or reduced activity of antioxidant mechanisms, plays a major role in the pathophysiology of heart failure.^6^ Increased ROS may further induce contractile, electrical, and structural remodeling in HF.^6^ Serum ROS level correlates with prognosis and severity HF.^34^ Reduction of oxidative stress is reported to improve inflammation, provide cytoprotectant effects, and ameliorate post-infarction remodeling.^35^ Adrenergic stimulation promotes ROS production by inducing a reductive redox as well as by stimulation of nicotinamide oxidases by β2-adrenergic activity.^36, 37^ Low-level vagal stimulation is reported to reduce ROS generation and improve antioxidant capacity in experimental models of myocardial infarction and heart failure.^38, 39^ Both antiadrenergic and cholinomimetic action of LLTS might also contribute to the in vivo inhibition of ROS production by augmentation of muscarinic activation(M2 receptors), anti-beta2 activity, downstream improvement of metabolic balance, as well as reduction of nicotinamide oxidase activity.^31, 34, 35^

### Limitations:

Our study carries several limitations, a small sample size being the first limitation. Second, the endpoints were focused on surrogate markers rather than clinical outcomes. However, this pilot study is hypothesis generating on the effects of LLTS on core pathogenic pathways in ADHF. Future prospective studies with large sample sizes are warranted to determine the effects of LLTS on clinical endpoints in ADHF. Third, although heart rate variability parameters are considered markers of cardiac autonomic activity, the effect of short-term LLTS on heart rate variability parameters has not been evaluated in our study. Our ongoing extended study (NCT02898181) of LLTS in acute heart failure (HFrEF and HFpEF) will further evaluate the effects on heart rate variability, inflammation, heart failure clinical biomarkers. In this ongoing study the duration of LLTS is now reduced to 2 hours/day.

## Conclusions

LLTS during hospital admission is safe in the acute decompensated heart failure patients with reduced ejection fraction and is associated with the mitigation of systemic inflammatory activity. Endothelial level oxidative stress activity, specifically cytosolic peroxide content, was also decreased with LLTS in ADHF, perhaps directly linked to attenuation in systemic inflammation. Further studies are underway to corroborate and extend these findings.

## Figures and Tables

**Table 1:** Serum cytokines and oxidative stress at admission and discharge in LLTS group*

Parameters		Admission	Discharge	*p*-value
**Serum cytokines (pg/mL)**	**IL-6**	7.42 (4.36–14.14)	0.89 (0.84–4.08)	**0.011**
	**CRP**	93.00 (35.55–112.75)	39.29 (24.23–55.84)	0.345
	**TNF-α**	18.14 (17.04–20.64)	14.42 (13.01–20.24)	0.483
	**sICAM1**	515.86 (352.86–580.02)	429.25(304.33–583.92)	0.792
	**sVCAM1**	1500.85(908.98–1569.83)	1304.01(1030.68–1457.31)	0.731
**Oxidative stress (fluorescein units)**	**DCF**	1363 (858.3–1689)	978.3 (752.7–1488)	**0.003**

* Value represented as median (IQR)

Abbreviation: CRP: C-reactive protein; DCF: Dihydrodichlorofluorescein; IL-6: Interleukin 6;TNF-α: Tumor necrosis factor α; sICAM1: Soluble intercellular adhesion molecule-1; sVCAM1: Soluble Vascular Cell Adhesion Molecule-1

**Table 2:** Serum cytokines and oxidative stress at admission and discharge in control group*

Parameters		Admission	Discharge	*p*-value
**Serum cytokines (pg/mL, except CRP-mg/dL)**	**IL-6**	27.54 (4.75–79.19)	30.36 (2.85–67.02)	>0.99
	**CRP**	62.93 (22.13–105.92)	48.74 (26.29–96.40)	0.912
	**TNF-α**	18.54 (15.78–21.93)	20.33 (16.72–24.90)	0.562
	**sICAM1**	307.13 (214.88–438.81)	341.66 (222.73–497.06)	0.633
	**sVCAM1**	1046.46(849.30–1539.96)	1174.59(1019.24–1587.435)	0.311
**Oxidative stress (fluorescein units**	**DCF**	1146 (982.3–1511)	1083 (864.8–1451)	0.094

* Value represented as median (IQR)

Abbreviation: CRP: C-reactive protein; DCF: Dichlorofluorescein; IL-6: Interleukin 6;TNF-α: Tumor necrosis factor α; sICAM1: Soluble intercellular adhesion molecule-1; sVCAM1: Soluble Vascular Cell Adhesion Molecule-1.

**Table 3:** Effect of LLTS on systemic inflammatory markers*

Serum cytokines	LLTS		Control		*p*-value^$^
	Baseline	% Change**	Baseline	% Change**	
**IL-6**	7.42 (9.78)	−78.48 (53.42)	27.54 (74.44)	−8.63 (32.80)	0.012
**CRP**	93 (77.21)	−12.69 (60.28)	62.93 (83.79)	6.74 (84.06)	0.549
**TNF-a**	18.14 (3.60)	−10.91 (41.46)	18.54 (6.14)	4.78 (31.98)	0.182
**sICAM1**	515.86 (227.16)	−2.82 (88.20)	307.13 (223.94)	2.29 (79.13)	0.720
**sVCAM1**	1500.85 (660.85)	−3.46 (45.17)	1046.46 (690.67)	19.38 (19.29)	0.400

* Value represented as median (IQR),

** : % changes of discharge-baseline value

$ p values between % change in LLTS and control group

Abbreviation: CRP: C-reactive protein; IL-6: Interleukin 6; TNF-α: Tumor necrosis factor α; sICAM1: Soluble intercellular adhesion molecule-1; sVCAM1: Soluble Vascular Cell Adhesion Molecule-1.
